# Internet of Things–Enhanced Mathematical Oncology: Conceptual Framework for Adaptive Cancer Care Modeling

**DOI:** 10.2196/73997

**Published:** 2026-07-13

**Authors:** Adeniyi Onasanya, Ramona Kyabaggu, Daryl Hepting

**Affiliations:** 1 Department of Computer Science, University of Regina, 3737 Wascana Parkway, Regina, SK, S4S 0A2, Canada, 1 306-351-5470; 2 Johnson Shoyama Graduate School of Public Policy, University of Regina, Regina, SK, Canada

**Keywords:** business process modeling, cancer care services, health care, health care informatics, health care systems, internet of things, IoT-based health care, mathematical modeling, mathematical oncology

## Abstract

The Internet of Things (IoT) is transforming various industries, including health care. IoT-based systems are increasingly prevalent in consumer health applications, while intelligent or smart devices equipped with sophisticated sensors are gaining recognition for their potential to improve clinical care practice and decision-making. Cancer care is a particularly promising area for IoT applications, enabling real-time and personalized interventions. However, empirical research on the effects of IoT in this field is limited due to the complexities inherent in cancer as a dynamic disease and the paucity of IoT-generated data available for research. This presents an opportunity to apply mathematical modeling to understand the effects of IoT under various scenarios. These analytical and “in silico” mathematical approaches are instrumental with limited data. Such models support the analysis of treatment uncertainty and patient response while balancing patient preferences, clinical outcomes, and system-level constraints. Grounded in mathematical oncology and health informatics, this paper proposes a conceptual framework that integrates real-time IoT data as dynamic inputs into adaptive mathematical models to simulate cancer dynamics. By exploring applications across multiple levels of analysis, the study demonstrates how IoT-enhanced mathematical models could inform implementation and optimize oncology services, addressing a critical gap in current research.

## Introduction and Motivation

The Internet of Things (IoT) represents a paradigm shift in technological advancement, enabling interconnected, intelligent, and responsive systems through continuous data collection, communication, and analysis. As a key driver of Industry 4.0, IoT facilitates the convergence of information technology and operational technology by interconnecting physical objects in internet-aware environments. Its rapid adoption across industries, governments, and society underscores its value in enabling interoperable, data-driven services beyond traditional machine-to-machine communication [1].

In health care, numerous IoT applications have been proposed to support care delivery, access, and patient safety [2-9]. However, despite growing interest, few studies empirically evaluate or mathematically model the effects of IoT interventions, particularly in cancer care. While mathematical oncology and IoT-based cancer care systems have mainly evolved in parallel, research that unifies these approaches remains limited. Integrating IoT with mathematical oncology offers an opportunity to link mechanistic disease dynamics with real-time, patient-specific data, enabling more adaptive and patient-centered cancer care.

The primary goal of this work is to investigate how integrating IoT technologies with mathematical oncology can enhance the modeling, analysis, and optimization of cancer care through real-time, data-driven, and adaptive approaches to improve treatment efficacy and patient outcomes. This study specifically builds on an established melanoma immunotherapy model [10] to develop a conceptual framework that integrates real-time IoT data into adaptive mathematical models of cancer dynamics and behavior. This framework extends treatment strategies and supports business process modeling by embedding IoT-enabled devices within business process modeling elements—tasks, events, activities, and resources—allowing continuous monitoring and evaluation of cancer conditions and characteristics in IoT-enabled care systems.

The paper is organized as follows. The *Overview and Literature* section reviews cancer, common treatment strategies, and existing mathematical models, highlighting their limitations. The *Case Study* section on immunotherapy for melanoma presents a conceptual framework for adaptive cancer care modeling using real-time IoT data as dynamic parameter inputs. The *Innovative Modeling Approach* section examines IoT-based health care applications in cancer care and integrates IoT with business process management to develop an end-to-end cancer care model. The *Implementation and Challenges* section discusses operational and security considerations for IoT-enabled cancer care systems. The paper concludes with key findings and directions for future research.

## Overview and Literature

### Cancer and Treatment Strategies

**Figure 1.**
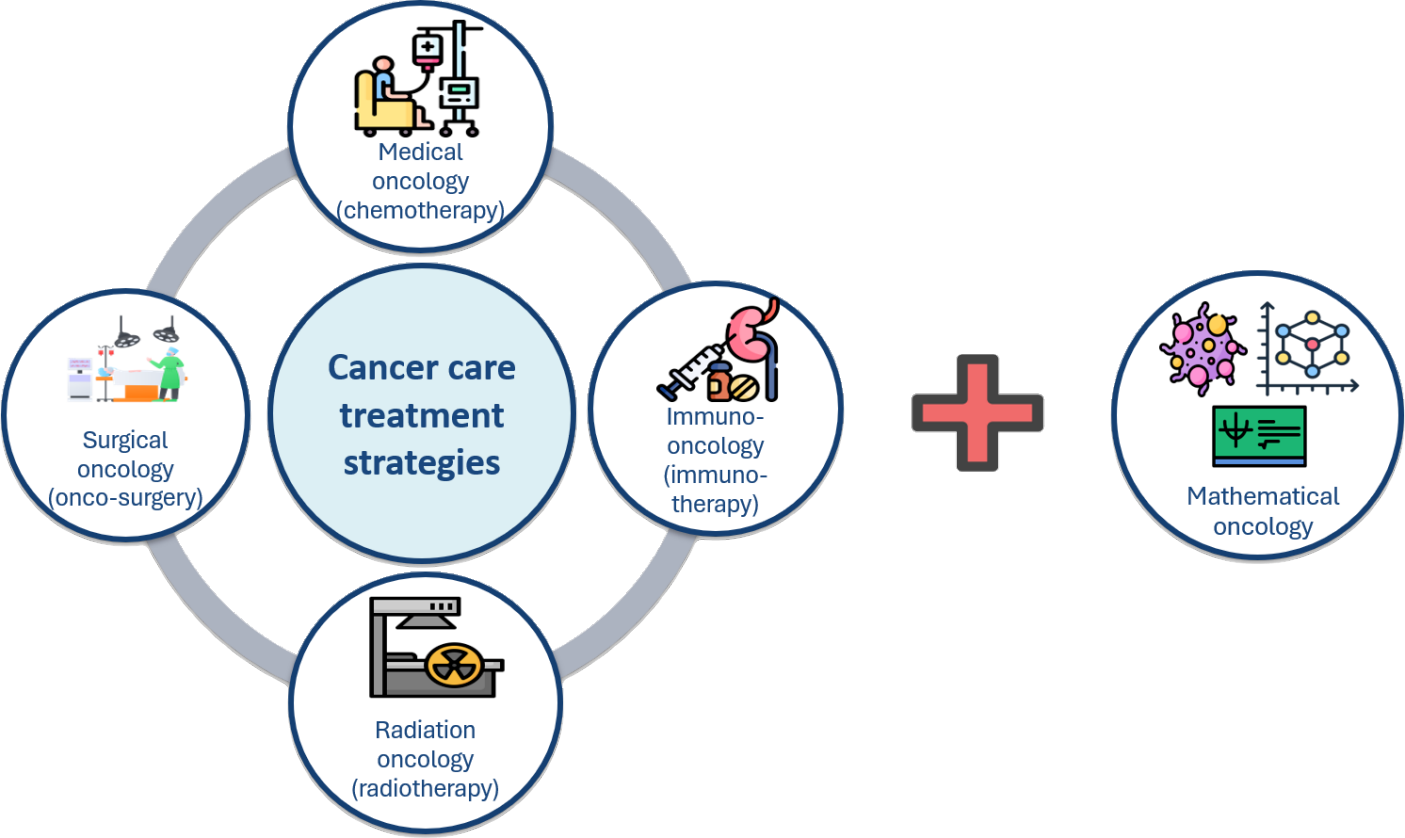
Cancer care treatment strategies and mathematical oncology.

Oncology is the medical discipline dedicated to the prevention, diagnosis, and treatment of cancer. Cancer results from genetic mutations that disrupt normal cellular regulation, leading to uncontrolled proliferation, tumor formation, tissue invasion, and potential metastasis [11]. Four primary clinical treatment modalities are widely used: onco-surgery, chemotherapy, radiotherapy, and immunotherapy, which are often applied individually or in combination, as illustrated in Figure 1.

Mathematical oncology is an interdisciplinary field that applies mathematical methods to study cancer dynamics and treatment outcomes, traditionally focusing on the responses of normal and cancerous cells to therapeutic inputs [6,12-16]. These models emphasize biological and physiological parameters—such as cell growth rates, population dynamics, invasion, and progression—to predict tumor growth, treatment response, and metastasis, thereby informing clinical decision-making.

With the rapid advancement of IoT in health care, integrating smart, connected devices into patient care enhances the practical relevance of these models. IoT-enabled mathematical modeling leverages real-time data from connected sensors to support adaptive, data-driven oncological practices beyond traditional mathematical oncology.

### Mathematical Modeling of Cancer Treatment

#### Overview

Cancer has a substantial global impact, with treatment outcomes varying widely due to pronounced patient and tumor heterogeneity, which complicates the prediction of disease progression. Cancer arises from genetic alterations driven by inherited predispositions, environmental exposures such as radiation and chemical carcinogens, and viral agents (deoxyribonucleic acid or ribonucleic acid viruses), leading to dysregulated cell cycles through oncogene activation and inactivation of tumor suppressors [17,18]. Mathematical oncology offers quantitative frameworks to improve prediction and support more effective treatment strategies.

A wide range of mathematical models has been developed to characterize tumor growth, treatment dynamics, and control mechanisms, particularly for chemotherapy, radiotherapy, and immunotherapy, though onco-surgery remains largely unaddressed. Growing evidence supports the use of mathematical modeling in cancer treatment, as patient-specific responses and tumor size dynamics challenge population-level, “one-size-fits-all” approaches [12,13,18-20]. Models of untreated tumor growth include linear, exponential, logistic, and sigmoidal forms such as Bertalanffy and Gompertz functions [20,21], while additional formulations describe immune-tumor interactions under immunotherapy, chemotherapy, and vaccine-based treatments [4,22].

#### Radiotherapy or Radiation Oncology

Liu and Yang [23] examined the dynamical behavior of normal cells under periodic radiation using mathematical models, deriving conditions for the persistence or extinction of normal and irradiated cells. Belostotski et al [13] proposed a Lotka-Volterra–based competition model capturing interactions between healthy and cancer cells under radiotherapy, incorporating multiple radiation control mechanisms, including “harvesting-type” control, which has been shown to produce spill-over effects on healthy cells that can be quantified to refine treatment strategies [13,19]. Weerasinghe et al [24] highlighted the importance of quantitative models in studying cancer cell plasticity, using them to inform therapeutic design and optimization across tumor progression stages such as avascular and vascular growth, angiogenesis, invasion, and metastasis within the tumor microenvironment.

#### Chemotherapy or Medical Oncology

Isaeva et al [16] use differential equation–based chemotherapy models to examine interactions among tumor, healthy, and immune cells, linking theoretical analysis with empirical evidence. Similarly, Song et al [10] propose a model that incorporates three cell populations and drug concentration to study immune-mediated tumor control and the side effects of chemotherapy on both tumor and effector cells. The dynamics of tumor and immune cell populations under chemotherapy are described by the following model [10]:


(1) $$\begin{array}{rl} N^{\prime }(t) & =aN(t)(1-bN(t))-\alpha_{1}N(t)T(t)-k_{N}u(t)N(t),\\ L^{\prime }(t) & =rN(t)T(t)-\mu L(t)-\beta_{1}L(t)T(t)-k_{L}u(t)L(t),\\ T^{\prime }(t) & =cT(t)(1-dT(t))-\alpha_{2}N(t)T(t)-\beta_{2}L(t)T(t)-k_{T}u(t)T(t),\\ u^{\prime }(t) & =v-\omega u(t) \end{array}\}$$

with initial conditions:

.$$N(0)=N_{0}§gt;0,\;L(0)=L_{0}§gt;0,\;T(0)=T_{0}§gt;0,\;\text{and }u(0)=u_{0}§gt;0$$

The variables *T*(*t*) and *N*(*t*) represent the population sizes of cancer cells and Natural Killer *NK* cells, respectively, at any given time *t*; *L*(*t*) for the cytotoxic tumor cell (CTL) population; and *μ*(*t*) for the amount of drug at the tumor site. The parameters in equation (1), along with their descriptions, are as depicted in Table 1.

**Table 1.** Description of the parameters in equation (1).

Parameter	Description	Parameter	Description
*𝑎*	Growth rate of NK^a^ cells	*β*_1_	CTL^b^ death rate
𝑏	Inverse of NK cells capacity	*β*_2_	Rate of CTL-induced tumor death
*c*	Growth rate of tumor	*ν*	Influx of drug
*d*	Inverse of tumor capacity	*ω*	Drug decay rate
*r*	Activation rate of CTLs	*k_N_, k_L_*	Immune cells killed by drug^c^
*μ*	CTL death rate	*k_T_*	Tumor cell killed by drug^c^
*α*_1_	NK cell death rate	—^d^	—
*α*_2_	Tumor death rate of NK	—	—

a NK: natural killer cell.

b CTL: cytotoxic tumor cell.

c Note that the 3 different response coefficients are denoted by *k_N_*, *k*_*L*_, and *k_T_* because chemotherapy affects all 3 cell populations and the model assumes side effects on both tumor and effector cells [10].

d Not applicable.

#### Immunotherapy or Immuno-Oncology

In immunotherapy, the effect of vaccine therapy is considered a parametric perturbation of the model commonly used in chemotherapy, in which simulations show that the efficiency of vaccine therapy depends on tumor size, the condition of the immune system, and the response of the organism to vaccination. The goal of immunotherapy is to enhance the antitumor resistance of an organism and improve the immune system’s condition [12]. There are 3 known main categories of immunotherapy: immune response modifiers (cytokines), monoclonal antibodies, and vaccines. Such immune response modifiers as interleukin-2 (IL-2), interferon-α (IFN-α), and tumor necrosis-α are widely used in cancer immunotherapy [10,12,23]. The system of equations alluded to in [10] for tumor-immune dynamics is based on the presence of treatment components consisting of 5 populations: tumor cells (*T*), cytotoxic tumor cell population (*L*), cytokine IL-2 population (*I*_2_), chemotherapeutic drug (*C*), and IFN-α population (*I*).


(2) $$\frac{dT}{dt}=-aT\ln\;\left(\frac{bT}{a}\right)-c(I)\;TL-M_{T}(I_{2})\left(1-e^{-C}\right)T$$

(3) $$\frac{dL}{dt}=d+eLI_{2}-fL-M_{L}(I_{2})\left(1-e^{-C}\right)L$$

(4) $$\frac{dI_{2}}{dt}=V_{I_{2}}(t)+\frac{gT}{T+l}-jLI_{2}-kTI_{2}$$

(5) $$\frac{dC}{dt}=V_{C}(t)-pC$$

(6) $$\frac{dI}{dt}=V_{I}(t)-qI$$

The tumor growth is described by Gompertzian law (the first term in equation 1). The destruction of tumor cells by *CTL* is presented by the second term in equation (1) in which the destruction rate is proportional to the number of tumor cells and *CTL* cell populations. In equation (3), *d* characterizes the steady inflow of *CTL* into the tumor site. The second and third terms in equation (2) describe CTL proliferation in response to IL-2 action and *CTL* death rate. In equations (3-5), 𝑉𝑖 (*I* = *I*_2_, *C*, *I*) describes the external influxes of IL-2, a chemotherapeutic drug, and IFN-α, respectively. Since therapy is assigned to a certain schedule, these influxes are taken to be time-dependent. IL-2 production in equation (3) is described by hyperbola (the second term), which allows us to consider a limitation in the stimulation of the immune system by the growing tumor. At small *T* (ie, *T* <= 𝑙), the growth rate is nearly linear in tumor size, while for big tumor (ie, *T*>>𝑙), it tends to a maximum constant value *g*. The parameter 𝑙 influences the IL-2 production rate. The smaller the value of 𝑙, the quicker the IL-2 production rate achieves its maximum value *g*.

#### Radiotherapy and Mathematical Oncology

Belostotski and Freedman [19] introduced a mathematical model for radiotherapy treatment strategy based on the Lotka-Volterra competition system, which was later extended by Liu and Yang [23]. The model assumes that cancerous and healthy cells coexist within the same region of the organism and that radiation preferentially eliminates cancer cells while exposing healthy cells to lower doses. Radiotherapy is thus modeled as a harvesting-type control mechanism that regulates the rate of change in both cancer and healthy cell populations, as discussed in Multimedia Appendix 1.

### Limitations of Mathematical Models

While mathematical models offer valuable frameworks for understanding cancer dynamics, several challenges limit their direct clinical applicability. Ghaffari et al [12] highlight a key gap between theoretical mathematical oncology and clinical practice, largely due to the limited availability of high-quality, patient-specific clinical data required for robust model validation. Song et al [10] similarly note that difficulties in accurately predicting treatment outcomes hinder practical implementation. Additional challenges include patient-level variability in parameter estimation, scarcity of comprehensive clinical datasets, reliance on simplifying assumptions that may not capture complex biological behaviors, and the difficulty of modeling treatment resistance. As noted by Yin et al [20], these factors introduce significant uncertainty into clinical use. In particular, critical parameters often derived from in vitro or in vivo experiments are difficult or impossible to measure directly, necessitating indirect estimation and inferential approaches. Collectively, these limitations constrain the routine clinical deployment of mathematical cancer models [25].

The study of complex IoT interventions in cancer care is constrained by the limited availability of IoT data, heterogeneity in care processes and interventions, and the intrinsic variability of cancer characteristics. Despite these challenges, mathematical models stand to benefit substantially from IoT integration, while modeling and simulation within IoT environments offer opportunities to mitigate many existing limitations. Incorporating additional data sources, such as nanotechnology-enabled sensing, and enabling real-time adaptation to diverse data streams can enhance predictive accuracy and clinical relevance. Although it has been suggested that the complete eradication of cancer cells is unattainable regardless of treatment type or timing [21], emerging evidence indicates that wireless sensor networks and IoT technologies can significantly improve treatment personalization and effectiveness, as demonstrated in our prior work [7]. Addressing data-related barriers—particularly IoT data governance, interoperability, quality, privacy, and security across the data lifecycle—is, therefore, essential for effective integration.

Overall, Yin et al [20] provide a comprehensive overview of mathematical models for tumor dynamics in cancer treatment, including empirical models of therapeutic effects, as well as algebraic, partial differential, stochastic, and deterministic formulations such as game-theoretic and integral-differential approaches. Building on this foundation, the models discussed in the preceding section—ranging from ordinary differential equations and tumor heterogeneity frameworks to biologically integrated tumor process models—inform the adaptation of these mathematical approaches to an IoT-enabled treatment context. This adaptation aims to strengthen computational and simulation capabilities, addressing the limitations of purely analytical models, as discussed in the following section.

### Patient and Clinician Adoption of IoT Interventions in Cancer Care

The adoption of the proposed approach in cancer care requires a supportive environment for change, including adequate resources and process redesign. Key resources include human, physical, network, and information assets, with patients and clinicians central to implementation and evaluation. Patient adoption is likely to be influenced by concerns about data privacy and security; targeted education, transparent data governance, user-centered design, and ongoing support can enhance trust, engagement, and adherence. Clinician adoption, on the other hand, depends on early involvement in system design to ensure alignment with clinical workflows and usability requirements. Although workload and technical complexity may pose barriers, the potential to streamline workflows, improve task integration, and optimize resource utilization can support clinician acceptance and sustained use.

The IoT intervention is intended to serve as a mathematical control mechanism in which IoT-aware parameters actively modify the system dynamics of the mathematical models. While the tumor-immune interaction equations are adapted from established mathematical oncology models, the proposed IoT-enhanced immunotherapy framework introduces a fundamental shift by enabling IoT-driven parameter adaptation. Unlike traditional IL-2 tumor-immune models that assume static or population-averaged parameters, the IoT-enhanced formulation incorporates time-varying, patient-specific parameter modulation informed by continuous physiological and biomarker sensing. This enables dynamic responses to toxicity, treatment tolerance, and disease progression, improving predictive stability and clinical relevance. The additional term introduced in the revised equations represents an IoT-driven feedback mechanism that adaptively modulates immune dynamics based on real-time patient data, extending classical fixed-parameter models into responsive, patient-specific systems. This formulation explicitly illustrates and justifies the role of IoT-driven modifications, as discussed in subsequent sections.

## Case Study (Immunotherapy for Melanoma)

### Overview

In demonstrating the impact of IoT integration in cancer care services, this study presents a case study based on an adapted immunotherapy mathematical model (equations 2-6) introduced in the *Overview of Cancer and Treatment Approaches* section. Immunotherapy was selected because such models are widely applied in melanoma treatment and leverage immune system stimulation to target cancer cells. Compared to other treatment modalities, immunotherapy requires continuous monitoring and dynamic management to mitigate adverse effects, making IoT-enabled devices particularly valuable for real-time patient monitoring and adaptive, data-driven care.

### Background

Melanoma patients often receive immunotherapy, which includes treatments with cytokines and immune checkpoint inhibitors. In this treatment option, the continuous monitoring and precise control of drug administration can significantly enhance the efficacy of such treatment therapies through the interfacing and integrating smart and intelligent connected devices.

### IoT Integration

1. Enabled (wearable) sensors: Patients wear IoT-enabled devices that monitor physiological parameters indicative of immune responses, such as body temperature, heart rate, and biomarker levels in sweat or blood.
2. Embedded (implantable) devices: Smart connected sensors are implanted near the tumor site to continuously measure tumor size and local immune markers directly.

This demonstrates the IoT integration for cancer treatment, which allows the enabled and embedded sensors and devices to be deployed in cancer care services by forming autonomous wireless ad hoc networks that interact with patients to collect information from patients and their environments. Through these ad hoc networks, the sensor nodes collaborate to perform the required task or tasks, such as processing, transmission, and aggregation of data from various sources, which are used to determine the nature of the illness by those in the circle of care to determine the nature of treatments and diagnoses to offer. As observed, data and information from sensors are transmitted or routed from various patient sites within the network to the data center through cloud services, facilitating the exchange of data and information from patient locations to the main hospital data center.

During implementation, various components are integrated to ensure the functioning of IoT-based systems or environments. Although these elements are broadly grouped at the component level, together they form an IoT-based component ecosystem. In practice, modeling an IoT-enhanced immunotherapy system for melanoma requires the integration, interconnectivity, and coordination of multiple components, including device, communication criteria, access technologies, communication network, data management, and application layers. Each component serves a distinct role in enabling effective operation and service delivery within the system.

The communication network component enables data sharing and traffic transmission from the point of observation to the data management platform and onward to various system applications. It operates through the access technologies component and supports communication across the network using routing protocols, different topologies and architectures, transmission channels, geographical locations, wireless sensor networks, wireless access points, and internet connectivity from the internet service provider, while ensuring security and encryption.

Therefore, adopting an appropriate routing strategy in wireless sensor networks is essential to balance optimality and efficiency while maintaining computation and communication performance [26]. This is especially important as health care resources become more interconnected and the number of sensor motes continues to grow. These motes collect sensory data by measuring, monitoring, and recording physical conditions in the health care environment and are connected through the internet to a central network infrastructure that enables data transmission and exchange, ultimately improving operational processes and service delivery.

### Proposed IoT-Enhanced Mathematical Framework

Mathematical model adaptation: The existing model equations can be adapted by introducing terms (ie, new variables) representing data inputs from IoT devices and adjusting treatment protocols based on real-time data.

The IoT-enhanced equations are derived from the baseline model by introducing IoT-driven input functions that adapt system dynamics in real time. For each added IoT function parameter, we provide corresponding biological and computational rationales to justify its inclusion in the mathematical framework.

Formula 1. Formula for tumor cell dynamics (*T*)


$$\frac{dT}{dt}=-aT\ln\left(\frac{bT}{a}\right)-c(I)\;TL-M_{T}(I_{2})\left(1-e^{-C}\right)T+S_{T}(t)$$

As shown in Formula 1, *S_T_*(*t*) represents the real-time tumor size adjustment based on data from the implantable or embedded sensors for its effects on immunotherapy.

- Biological rationale: Tumor burden evolves heterogeneously and may change rapidly in response to immunotherapy. Implantable or embedded sensors (eg, tumor microenvironment probes, imaging-derived biomarkers) can provide near-real-time indicators of tumor growth, hypoxia, or metabolic activity that are not captured by fixed growth or killing rates.
- Computational rationale: The term *S_T_*(*t*) introduces a corrective adjustment to tumor dynamics based on observed tumor-state deviations. Computationally, this term allows the tumor growth trajectory to be continuously recalibrated using sensor-derived feedback, improving model responsiveness and reducing reliance on static growth assumptions.

Formula 2. Formula for CTL dynamics (*L*):


$$\frac{dL}{dt}=d+eLI_{2}-fL-M_{L}(I_{2})\left(1-e^{-C}\right)L+S_{L}(t)$$

As shown in Formula 2, *S_T_*(*t*) represents the CTL modulation influenced by wearable sensor data for its effects on immunotherapy.

- Biological rationale: CTL activity is sensitive to systemic stress, fatigue, and immune suppression, which can be indirectly inferred from wearable sensor data such as heart rate variability, temperature, or activity levels. These physiological signals correlate with immune competence and treatment tolerance.
- Computational rationale: The term *S_T_*(*t*) enables time-varying modulation of CTL population dynamics in response to the patient’s state. This prevents the unrealistic persistence of immune efficacy under adverse conditions and allows the model to reflect immune exhaustion or recovery observed clinically.

Formula 3. Formula for IL-2 Dynamics (*I*_2_):


$$\frac{dI_{2}}{dt}=V_{I_{2}}(t)+\frac{gT}{T+l}-jLI_{2}-kTI_{2}+S_{I_{2}}(t)$$

As shown in Formula 3, $S_{I_{2}}(t)$ represents an adjustment in IL-2 based on continuous biomarker monitoring for its effects on immunotherapy that is, a function of a sensor-derived toxicity or inflammation signal *z*(*t*).

To illustrate the qualitative effect of IoT integration on immunotherapy dynamics, we introduce a normalized sensor-derived control signal *z*(*t*) ∈ [0,1] representing a composite patient toxicity and immune-activation index. This signal, computed from continuously monitored physiological and biomarker data (eg, body temperature, heart rate variability, oxygen saturation, and inflammation-related markers), drives the IoT feedback term $S_{I_{2}}(t;z(t))$ in the IL-2 dynamics. The term $S_{I_{2}}(t;z(t))$ enables real-time, patient-specific modulation of immune response, transforming the model from a static, open-loop formulation into an adaptive, feedback-driven system. This enhances predictive stability and clinical plausibility by reflecting treatment adjustments informed by continuous monitoring rather than fixed schedules.

- Biological rationale: IL-2 levels are tightly linked to immune activation and toxicity, with excessive IL-2 associated with severe adverse effects. Continuous biomarker monitoring (eg, cytokine levels, inflammatory markers) provides early signals of immune overactivation or suppression.
- Computational rationale: The IoT-driven $S_{I_{2}}(t)$ acts as an adaptive feedback mechanism that adjusts IL-2 dynamics in real time. This allows the model to represent dose adjustment, treatment delay, or attenuation strategies triggered by early biomarker changes, improving stability and clinical plausibility.

Formula 4. Formula for drug dynamics (*C*):


$$\frac{dC}{dt}=V_{C}(t)-pC+S_{C}(t)$$

As shown in Formula 4, *S_C_*(*t*) represents the chemotherapeutic drug infusion rates controlled by IoT devices to maintain optimal drug levels for its effects on immunotherapy.

- Biological rationale: Chemotherapeutic and immunotherapeutic drug infusion rates are increasingly controlled by smart pumps and IoT-enabled delivery systems that respond to patient-specific tolerance and toxicity signals.
- Computational rationale: The term *S_C_*(*t*) converts drug administration from a predefined input into a sensor-driven control variable. This enables dynamic regulation of drug concentration to maintain therapeutic efficacy while minimizing toxicity, reflecting real-world adaptive dosing practices.

Formula 5. Formula for IFN-α dynamics (*I*):


$$\frac{dI}{dt}=V_{I}(t)-qI+S_{I}(t)$$

As shown in Formula 5, *S_I_*(*t*) represents the IFN-α level adjustments based on sensor data for its effects on immunotherapy.

- Biological rationale: IFN-α plays a central role in immune activation but is also associated with systemic side effects. Sensor-derived indicators such as fever, fatigue, and inflammatory response provide indirect measures of IFN-α activity and tolerance.
- Computational rationale: The IoT term *S_I_*(*t*) allows IFN-α dynamics to be adaptively adjusted based on patient response, preventing sustained overactivation and enabling more realistic immune regulation within the model.

### Implementation

- *Real-time data processing*: IoT devices transmit data to a central system where they are processed in real time. The system uses algorithms to predict and adjust the influx rates $\left(V_{I_{2}}(t),\;V_{C}(t),\;V_{I}(t)\right)$ and introduces corrections $\left(S_{T}(t),\;S_{L}(t),\;S_{I_{2}}(t),\;S_{C}(t)\;and\;S_{I}(t)\right)$ based on the patient’s current state and treatment response.

- *Feedback system*: The treatment regimen is adjusted dynamically, allowing for personalized therapy schedules that optimize immune response and tumor suppression based on continuous feedback.

### Conclusion

Integrating IoT in immunotherapy allows for dynamic, personalized treatment adjustments that can potentially lead to better patient outcomes by ensuring optimal drug levels and immune responses at all times. This approach leverages the power of real-time data to make informed decisions on the complex dynamics of cancer treatment.

### Definitions of Parameters

The mathematical modeling of oncology treatment pathways and real-time patient monitoring relies on a specialized set of variables, which are categorized into biological parameters, IoT input functions, and time-dependent external influxes

1. Biological parameters:
  - Tumor growth parameters (*a* and *b*): They reflect the natural growth rate and carrying capacity of the tumor.
  - Immune response modifiers (*c*(*I*), *M_T_* (*I*_2_), and M*_L_*(*I*_2_)): They depend on the specific immune response elicited by different cytokines or drugs.
  - Drug effectiveness (*e*^-^*^c^*): It represents the effectiveness of the chemotherapeutic drug, typically modeled as an exponential decay.
  - *CTL* dynamics (*d, e,* and *f*): They represent the rate of *CTL* inflow to the tumor, *CTL* proliferation rate, and death rate, respectively.
  - Production and decay rates (*g, j, k, p,* and *q*): They control the dynamics of *IL-2*, *CTL*, and drug concentrations.
  - I: It affects the production rate of IL-2 by the tumor.
2. IoT input function parameters:
  - Sensor data adjustments $\left(S_{T}(t),\;S_{L}(t),\;S_{I_{2}}(t),\;S_{C}(t),\;\text{and }S_{I}(t)\right)$: They are model functions that adjust parameters based on real-time data from IoT devices.
3. External influxes:
  - Time-dependent influxes $\left(V_{I_{2}}(t),\;V_{C}(t),\;V_{I}(t)\right)$: They define how external treatments are administered over time.

## Innovative Modeling Approach to Cancer Care Through IoT

### Overview

Cancer research is increasingly moving toward intelligent, IoT-enabled health care delivery for cancer care [1,5-7,22]. Integrating IoT technologies with mathematical modeling enhances the precision of treatment dynamics by enabling the adaptive control of therapy parameters and patient-specific responses. This convergence gives rise to a mathematically modeled IoT-based cancer care system that embeds control mechanisms, parameters, and behaviors within IoT-driven clinical workflows.

Simulation and computational approaches can effectively bridge the gap between the theoretical frameworks of mathematical oncology and clinical practice. Central to these models are mathematical equations, especially differential equations, which quantify the behaviors and relationships within biological systems [12,16,17]. These models use specific biological parameters, such as growth rates or drug responses, enabling the simulation of diverse scenarios. Real-time data integration allows for further dynamic adjustments to parameters in response to individual-level factors or conditions. Overall, the responsiveness and dynamism of computational and simulation-enhanced mathematical models support the rapid validation of IoT-enabled cancer treatment effects under different scenarios.

The complex modeling required to validate the effects of IoT-enabled cancer treatment underscores the need for approaches that are both responsive and informed by underlying mechanisms. These models transcend the limitations of data reliance typical of population-level statistical methods, including some computational machine-learning techniques. Instead, they use mathematical models that clarify dynamic causal mechanisms between individuals, supplemented by the best available data, thereby enhancing predictive accuracy for personalized medicine.

In oncology, mathematical modeling approaches such as agent-based models and cellular automata are increasingly used [12]. These models are pivotal in understanding the biological complexities of cancer and developing effective therapies. When considering the application and implementation of IoT technologies, methods such as process simulation and digital models offer another dimension through which mathematical oncology can inform and optimize health system operations. These approaches provide advanced computational techniques to enhance the efficiency and effectiveness of service delivery and overall management within health care systems, ensuring a comprehensive and integrated approach to both individual patient care and broader health system settings.

By integrating IoT and related technologies, we can address many challenges, such as resistance and assumptions, that have hindered the effectiveness of various models in clinical trials and routine patient care. This integration enhances personalized approaches, helping to pinpoint the most effective treatment options and therapeutic regimens. Additionally, the use of advanced sensors, such as nanodevices implanted in patients, allows for the real-time monitoring of cancer’s nature and progression. Coupled with intelligent medical image analysis and the application of 3D computer-interactive technologies in virtual reality, augmented reality, and mixed reality contexts, these innovations offer significant potential to improve cancer treatment and the patient’s health care experience more effectively and efficiently [3].

Data sharing in health care environments, particularly with IoT-generated big data, presents a challenge due to the diversity of smart connected sensors, objects, devices, data, services, and other supporting applications resulting from the real-time flow and volume of data between systems. The adoption of health care standards and protocols for data interoperability, such as Health Level 7 connectivity, Digital Imaging and Communications in Medicine, and Fast Healthcare Interoperability Resources, may be critical to facilitating appropriately scaled and seamless data exchange between systems. In contrast, conformance to privacy and security standards and policies, in addition to these technical standards, can help protect highly sensitive personal health information from privacy risks and incidents.

### Applications of IoT-Based Health Care System for Cancer Care

#### Overview

**Figure 2.**
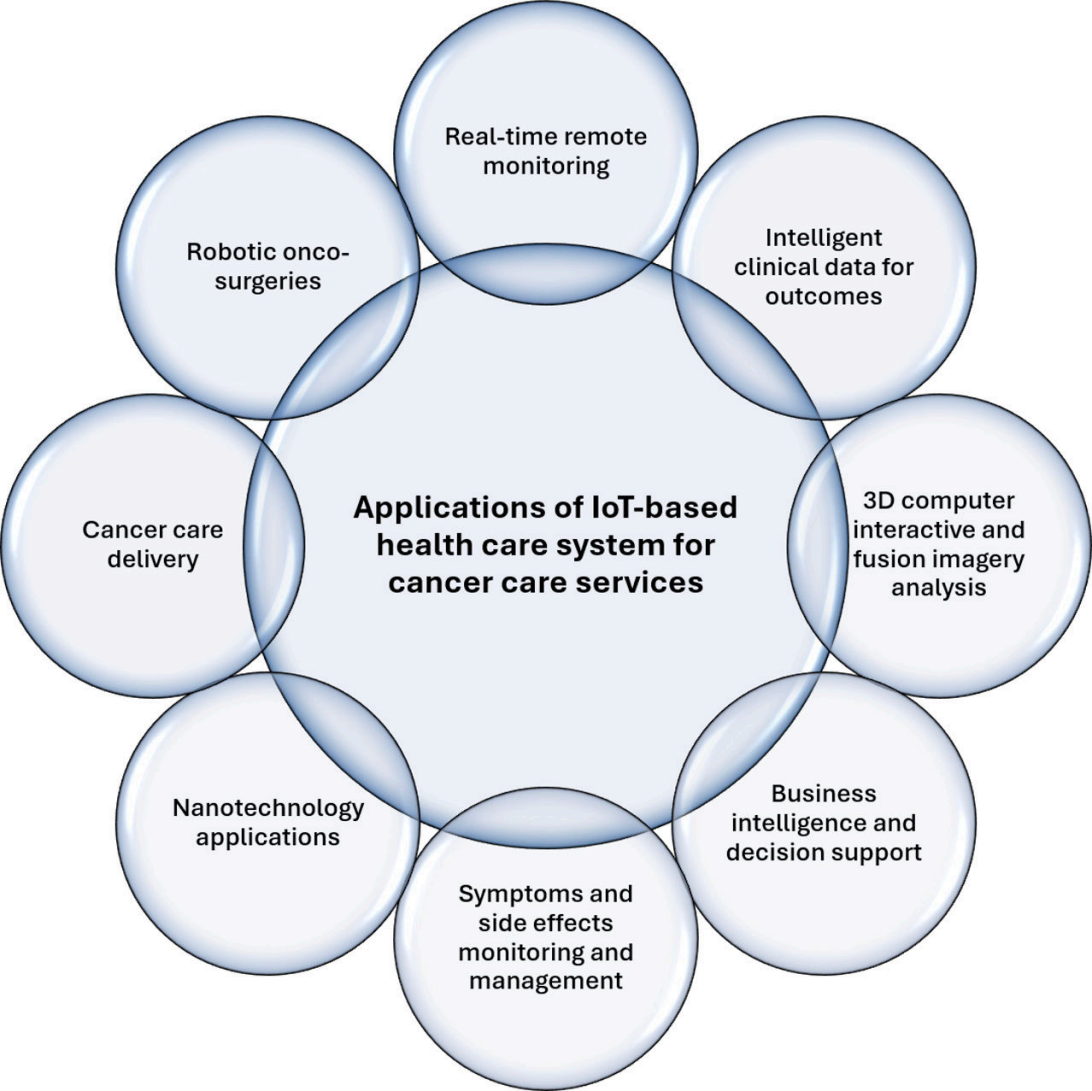
Applications of Internet of Things (IoT)–based health care system for cancer care.

Mathematical biological parameters describing cancer growth, cell populations, invasion, and progression are integrated into IoT-based cancer care services to enhance the design, simulation, and deployment of integrated health care solutions. The convergence of IoT and related technologies enables novel, data-driven approaches to cancer care. Figure 2 illustrates key IoT-based health care applications for cancer care.

#### Real-Time and Remote Monitoring

IoT devices are seamlessly integrated with cancer treatment protocols to enable real-time and remote monitoring of patients, including the capture and exchange of vital signs, patient-reported measures, activity, and biometric data across various geographical locations, enabled by IoT-connected technologies.

#### Intelligent Clinical Data for Outcomes

Integrating IoT-related technologies into cancer care facilitates a significant shift from traditional to digital health care. The electronic health record is anticipated to help manage this data stream, characterized by an expanded volume and variety of quality data for routine use in value-based learning health systems. These capabilities, not offered by IoT modeling approaches alone, support comprehensive patient management, including communication, monitoring, alerting, diagnosis, prognosis, and treatment. However, streams of personal data collected from IoT-connected devices carry ethical implications. Ethical guidelines and regulations for cancer patient data must be established to safeguard patient data against hacking, compromise, misuse, misdiagnosis, unauthorized access, attacks, and breaches, while improving transparency and accountability.

#### 3D Computer Interactive and Fusion Imagery Analysis

Intelligent medical image analysis using 3D computer interactive and fusion imagery technologies will significantly improve cancer treatment, especially in personalized health care experiences, making treatment more effective and efficient.

#### Business Intelligence and Decision Support

IoT-based cancer care provides a framework for analyzing patient data from IoT-enabled devices through business analytics. This assists health care providers and clinical researchers in converting data streams about patients’ health conditions into actionable insights and evidence-based decision-making at all levels.

#### Symptoms and Side Effects Monitoring and Management

Systemic cancer treatment may lead to significant side effects and complications such as allergies, side effects, and other adverse effects. Current mathematical modeling approaches do not account for these variances, but IoT platforms provide an interface to collect data from various devices and analyze them to detect anomalies. This capability is essential for rapid diagnosis and treatment adjustments, thereby effectively improving the quality of life of patients with cancer by managing these issues.

#### Nanotechnology Applications

The integration of IoT with nanotechnology (such as nanomaterials, nanodevices, nanostructures, nanoparticles, nanoprocessors, and nanonetworks) and communication engineering has led to the development of the Internet of Nano-Things and the Internet of Bio-Nano Things. These platforms facilitate interconnectivity, automation, and data exchange by embedding biosensors and computing nanodevices in biological systems, thereby enhancing cancer treatment through precise monitoring and targeted therapy delivery.

#### Cancer Care Delivery

Integrating IoT technologies with oncological treatment strategies changes how cancer care is administered by providing more responsive, personalized, and accessible treatment experiences that promote patient survival and quality of life. IoT devices, including smart sensors, enable the continuous monitoring of cancer evolution, optimizing treatment plans, and minimizing exposure of healthy tissues to harmful effects. In addition, IoT collaborative delivery models facilitate real-time communication and coordination among health care providers and empower patients by providing access to their health data for shared decision-making.

#### Robotic Onco-Surgeries

Integrating IoT with surgical robotics enables precise onco-surgical procedures by supporting high-accuracy, time-intensive interventions under continuous monitoring. IoT-enabled robots, augmented with embedded artificial intelligence, enhance surgical precision in targeting and excising locoregional tumors, including lymphadenectomy, while preserving healthy tissue within clinical margins. Real-time IoT monitoring during and after surgery supports patient stability, surgical condition awareness, and coordinated management of drains and tubes, thereby improving patient safety and postoperative recovery.

### Integrated IoT-Based Cancer Care System Through Modeling Tools

Cancer care services stand to benefit substantially from IoT-enabled technologies; however, as IoT technologies enable more personalized oncology treatment strategies, mathematical frameworks must evolve to incorporate the advancements made possible by IoT. Mathematical oncology provides the foundation for IoT-adapted models that support drug delivery optimization, predictive patient monitoring, treatment scheduling, and interpretation of complex sensor-generated data streams. This integration ensures that IoT applications in oncology are clinically relevant and operationally feasible.

**Figure 3.**
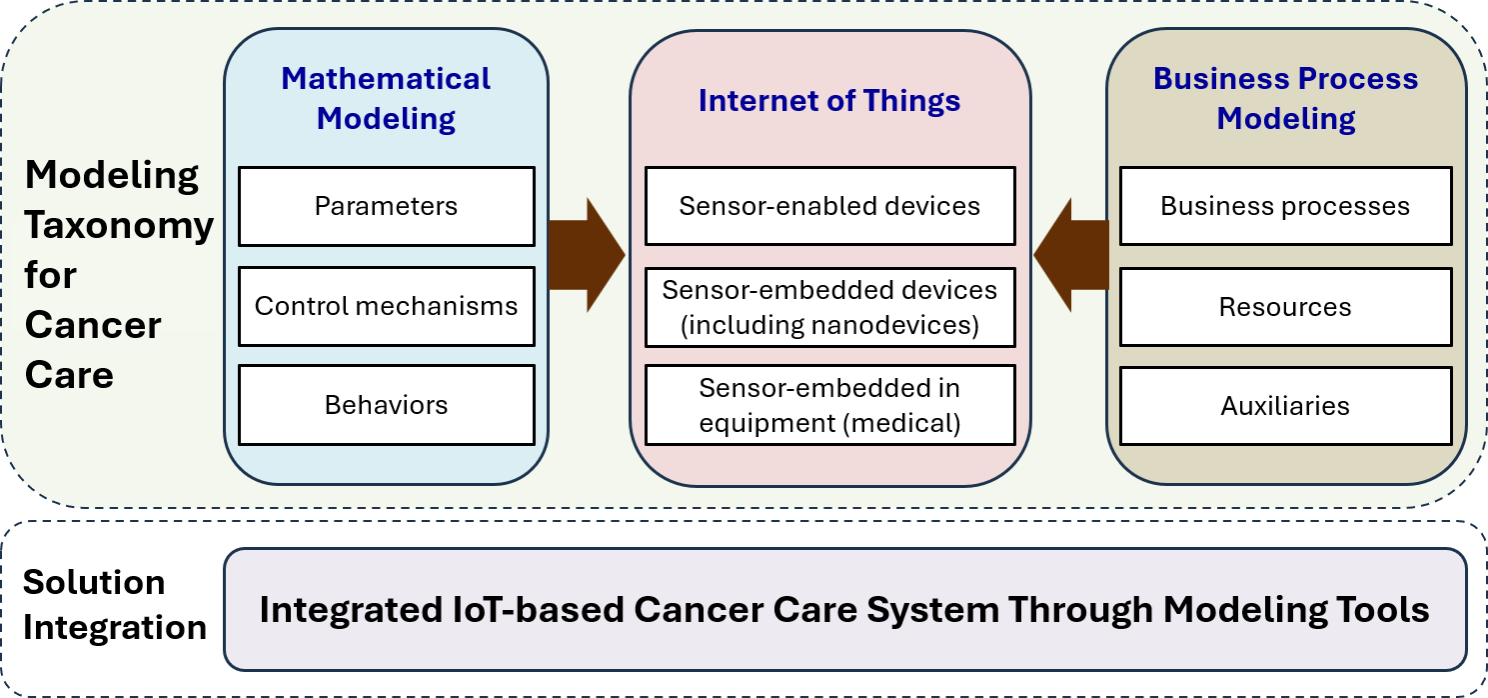
Taxonomy of Modeling tools of an integrated Internet of Things (IoT)–based cancer care system.

With continued advances, integrating IoT, mathematical modeling, and complementary analytical tools supports more automated and adaptive cancer care services, informing workflow redesign, clinical decision support, and improved care delivery. Figure 3 illustrates the taxonomy and interrelationships of cancer care modeling approaches examined in this study and future work, showing how IoT-enabled devices are embedded within business process modeling artifacts—tasks, events, activities, and resources—to enable the continuous monitoring of cancer conditions and characteristics.

Business process management (BPM) tools are widely used in health care due to their service-oriented nature [27,28]. In cancer care, BPM provides a structured approach to evaluate IoT-enabled services, tasks, and workflows, while IoT enhances efficiency and service delivery. BPM modeling helps identify redundancies, inefficiencies, and gaps in current processes, guiding the design of improved or future processes or workflows that better support the circle of care. It also informs optimal resource allocation and effective IoT interventions to optimize processes and improve patient outcomes. IoT-enabled BPM enables real-time monitoring and analysis of patient conditions and treatment responses using on-body, in-body, and off-body devices, supporting proactive and adaptive care.

Incorporating IoT into health care systems for cancer care services, especially radiotherapy, has been revolutionizing. A recent report by Chen et al [2] suggests that radiotherapy has evolved as a frontier discipline in cancer care services, driven by innovative advancements in artificial intelligence, which extend human intelligence. This has resulted in the widespread implementation of “intelligent radiotherapy," encompassing various tools, namely, big data, deep learning, extended reality, digital twin, radionics, and the IoT. Recognizing that target contouring and treatment planning are at the heart of the entire radiotherapy process, it becomes clear that there is considerable potential for improvement in this area. Radiation oncologists can begin to enhance the radiotherapy process with intelligent radiotherapy that facilitates automation and intelligence, especially through business process modeling. This demonstrates that when evaluating effects, we cannot overlook the significance of IoT and related digital technologies in the modern health care system landscape.

All these applications and technologies, through smart and intelligent devices and equipment, are adopted and adapted to underpin smart cancer care service delivery, which supports the conclusion that cancer treatment under a combination of multiple treatment strategies will yield better outcomes. This extends even to the use of the mathematical treatment strategies or model-based approaches. Drawing from the assertion by Ghaffari et al [12] that “the field of mathematical oncology is somewhat disconnected from the clinical practice of oncology,” we cannot apply mathematical modeling with only traditional oncology treatments in mind; instead, we must adapt to model the complex interventions that are enhanced by technological advancements.

## Implementation and Challenges

It is essential to address the technological, regulatory, operational, and security challenges associated with implementing IoT-enabled cancer care systems within the proposed modeling framework. Mathematical and business process simulations offer a practical means or mechanism to translate or operationalize compliance, security, interoperability, and reliability requirements into testable parameters, enabling their evaluation before system deployment.

From a business process perspective, cancer-care workflows (eg, monitoring, alert generation, clinical decision, and intervention) can be modeled using discrete-event or business process model and notation-based simulations in which network latency, data loss, authentication delays, interoperability failures, or device downtime are injected as stochastic disturbances. These perturbations propagate through the workflow as delayed alerts, missed detections, or increased clinician response times, allowing the system to quantify process-level degradation such as time-to-intervention, queue buildup, or alarm fatigue under varying operational conditions.

From a mathematical modeling perspective, these process-level delays can be directly coupled to treatment-response models (eg, tumor-immune interaction models). For example, latency in vital-sign transmission or intermittent sensor dropout can be represented as delayed or incomplete feedback signals, leading to mistimed dose adjustments or prolonged exposure during adverse events. Simulation enables the quantification of the resulting clinical impact, such as reduced dose intensity, increased toxicity risk, or diminished therapeutic response.

Together, this integrated simulation framework functions as a predeployment environment, enabling the identification of critical thresholds—such as maximum allowable latency, minimum data completeness, or minimum sampling rates under low-power constraints—beyond which safety or efficacy is compromised. These findings can be translated into evidence-based design requirements that guide decisions on edge analytics, redundancy, interoperability standards, and security architectures, thereby influencing the performance of IoT-enabled devices and sensors.

## Discussion and Future Work

This research proposes an integrated framework that combines mathematical modeling and IoT-based interventions to enhance cancer care delivery. Drawing on concepts from mathematical oncology, the study explores selected models to examine treatment dynamics while acknowledging the broader landscape of existing cancer models and their associated limitations. These limitations—particularly data scarcity, uncertainty, and system complexity—motivate the incorporation of IoT technologies to balance the trade-offs among patient preferences, clinical outcomes, and health care system constraints. An adaptive, innovative IoT-enabled modeling approach has been developed and demonstrated through a melanoma immunotherapy case study, highlighting the potential of real-time sensing and adaptive decision support. Data interoperability is identified as a key challenge, necessitating the use of health care data standards and secure protocols to enable real-time data exchange while preserving privacy and security.

Future research will advance this work along two complementary directions: mathematical modeling and business process modeling. From a mathematical perspective, future studies will incorporate additional parameters and empirically derived patient data to refine and validate treatment-response models. From a business process perspective, IoT-enabled cancer-care workflows will be simulated to assess performance, scalability, and regulatory compliance across different levels of oncology service delivery.

The integration of these perspectives offers a pathway toward digitally transformed cancer care, enabling the systematic evaluation of both clinical and operational capabilities across the continuum of care—from screening and diagnosis to treatment administration, rehabilitation, and follow-up. Thus, this disrupts current health care practices by replacing traditional health care with a frontier of digital health care. Methodologically, this will involve structured business process modeling using BPMN 2.0 (by means of mathematical modeling of cancer care services), application of the BPM lifecycle to transition from as-is to to-be care pathways, and simulation of IoT-enabled future-state models. Together, mathematical and process-based simulations provide a rigorous foundation for improving care quality, supporting decision-making, and guiding the evolution of IoT-enabled cancer care systems.

## Supplementary material

10.2196/73997 Multimedia Appendix 1 A system of radiation treatment models based on competitive Lotka-Volterra dynamics and Manley’s asymmetrical division framework.

## Abbreviations

BPM: business process management
CTL: cytotoxic tumor cell
IFN-α: interferon-α
IL-2: interleukin-2
IoT: Internet of Things

## References

[1] Onasanya A, Lakkis S, Elshakankiri M (2019). Implementing IoT/WSN based smart Saskatchewan Healthcare System. Wireless Netw.

[2] Chen G, Cui J, Qian J (2022). Rapid progress in intelligent radiotherapy and future implementation. Cancer Invest.

[3] Dimitrov DV (2016). Medical internet of things and big data in healthcare. Healthc Inform Res.

[4] Riazul Islam SM, Humaun Kabir M, Hossain M (2015). The internet of things for health care: a comprehensive survey. IEEE Access.

[5] Onasanya A, Elshakankiri M IoT implementation for cancer care and business analytics/cloud services in healthcare systems.

[6] Onasanya A, Elshakankiri M Secured cancer care and cloud services in IoT/WSN based medical systems.

[7] Onasanya A, Elshakankiri M (2021). Smart integrated IoT healthcare system for cancer care. Wireless Netw.

[8] Yin Y, Zeng Y, Chen X, Fan Y (2016). The internet of things in healthcare: an overview. J Ind Inf Integr.

[9] Chatbot App.

[10] Song G, Liang G, Tian T, Zhang X (2022). Mathematical modeling and analysis of tumor chemotherapy. Symmetry.

[11] Evan GI, Vousden KH (2001). Proliferation, cell cycle and apoptosis in cancer. Nature.

[12] Ghaffari Laleh N, Loeffler CML, Grajek J (2022). Classical mathematical models for prediction of response to chemotherapy and immunotherapy. PLoS Comput Biol.

[13] Belostotski G (2005). A control theory model for cancer treatment by radiotherapy [Master's thesis]. https://ualberta.scholaris.ca/server/api/core/bitstreams/62ac2fde-2dd8-49a2-b4d4-e61efd8dac2f/content.

[14] Brady R, Enderling H (2019). Mathematical models of cancer: when to predict novel therapies, and when not to. Bull Math Biol.

[15] Enderling H, Alfonso JCL, Moros E, Caudell JJ, Harrison LB (2019). Integrating mathematical modeling into the roadmap for personalized adaptive radiation therapy. Trends Cancer.

[16] Isaeva OG, Osipov VA (2009). Different strategies for cancer treatment: mathematical modelling. Comput Math Methods Med.

[17] Kuang Y, Nagy JD, Eikenberry SE (2016). Introduction to Mathematical Oncology.

[18] Luo MC, Nikolopoulou E, Gevertz JL (2022). From fitting the average to fitting the individual: a cautionary tale for mathematical modelers. Front Oncol.

[19] Belostotski G, Freedman HI (2005). A control theory model for cancer treatment by radiotherapy. Int J Appl Mech.

[20] Yin A, Moes D, van Hasselt JGC, Swen JJ, Guchelaar HJ (2019). A review of mathematical models for tumor dynamics and treatment resistance evolution of solid tumors. CPT Pharmacometrics Syst Pharmacol.

[21] Manley O (2014). A mathematical model of cancer networks with radiation therapy. J Young Investig.

[22] 5 IoT applications in healthcare field you must know. DataFlair.

[23] Liu Z, Yang C (2014). A mathematical model of cancer treatment by radiotherapy. Comput Math Methods Med.

[24] Weerasinghe HN, Burrage PM, Burrage K, Nicolau DV (2019). Mathematical models of cancer cell plasticity. J Oncol.

[25] Vieira LC, Costa RS, Valério D (2023). An overview of mathematical modelling in cancer research: fractional calculus as modelling tool. Fractal Fract.

[26] Sohraby K, Minoli D, Znati T (2007). Wireless Sensor Networks Technology, Protocols, and Applications.

[27] Rolón E, Chavira G, Orozco J, Soto JP (2015). Towards a framework for evaluating usability of business process models with BPMN in health sector. Procedia Manuf.

[28] Sari MP, Pratama NR, Dachyar M Application Internet of Things (IoT) in healthcare to optimize waiting time using the business process reengineering (BPR) approach. https://ieomsociety.org/proceedings/2022istanbul/820.pdf.

