# Supplementary material for: Internet of Things–Enhanced Mathematical Oncology: Conceptual Framework for Adaptive Cancer Care Modeling
Source: JMIR Cancer. 2026 Jul 13;12:e73997. doi: 10.2196/73997 (PMC13361616; doi:10.2196/73997)
Supplement: Multimedia Appendix 1 [file cancer-v12-e73997-s001.docx]

**Supplementary material**

#### **Radiotherapy + Mathematical Oncology**

Radiotherapy is thus modelled as a harvesting-type control mechanism that regulates the rate of change in both cancer and healthy cell populations, as discussed below.

Let $x_{1}$(𝑡) be the concentration of healthy cells, and let $x_{2}$(𝑡) be the concentration of cancer cells. Then, the model as proposed in [18] takes the form:

$\begin{matrix} \dot{x}_{1}=\alpha_{1}x_{1}\left( 1-\frac{x_{1}}{k_{1}} \right)-\beta_{1}x_{1}x_{2}-\epsilon D\left( t \right)x_{1}; \end{matrix}$ (1)

$\begin{matrix} \dot{x}_{2}=\alpha_{2}x_{2}\left( 1-\frac{x_{2}}{k_{2}} \right)-\beta_{2}x_{1}x_{2}-D(t)x_{2}; \end{matrix}$ (2)

where $\dot{x}=\frac{dx}{dt}$ and $D\left( t \right)$=strategy of the radiotherapy and $x_{i}(t)\geq0,i=1,2$, are the concentrations of healthy and cancer cells, respectively.

We assume that $D(t)=\gamma>0$ when $t\in[nw,nw+L)$ (treatment stage) and $D\left( t \right)=0$ when $t\in[nw+L,(n+1)w]$ (no treatment stage, which implies recovery stage) for all $n=0,1,2$,…, where w is the periodic of treatment and $0<L<w$ is the radiation treatment time.

During the process of cancer radiation treatment, healthy cells are also affected. The proportion of the radiation is $\epsilon D(t)$, $\epsilon>0$ ($\epsilon=0$is the ideal, but impossible to achieve in a practical scenario). $\alpha_{i}>0$ (i=1, 2) are the respective carrying capacities, and $\beta_{i}>0$ (i=1, 2) are the respective competitive coefficients.

In the absence of radiation, cancer (i.e. $x_{2}$) takes over resulting in the following conditions: $k_{1}<\frac{\alpha_{2}}{\beta_{2}}$ and $k_{2}>\frac{\alpha_{1}}{\beta_{1}}$. During the treatment stage, the radiation harvesting amount is $\gamma x_{2}$to the cancer cells and $\epsilon\gamma x_{1}$to the healthy cells.

Conversely, the mathematical modelling approach adopted by Manley [21] for cancer treatment focuses on using linear cancer networks, where the number of cells grows linearly concerning time *t*, as shown below. This is viewed as having a slower growth rate than the other cancerous network types. Linear cancer networks begin with one type of cancerous cell (hereafter *A* cell). When *A* cells divide into two cells, they produce another *A* cell and an *A* cell that differentiates into another type of cell (hereafter *B* cell). Then *A* Cells are cancer stem cells while *B* cells are terminal (which means they do not divide).

This asymmetrical division results in the number of *A* cells staying constant and the number of *B* cells growing linearly. In this case, *A* cells are the cancerous cells because they are responsible for producing the unlimited growth of *B* cells, and the *B* cells are not necessarily cancerous because they do not produce growth. This results in a tumour that consists of mostly *B* cells but is sustained by the *A* cells. The mathematical model is a basic linear cancer network model consisting of calls type *A* and type *B*.

A system where the population of *A* cells stayed constant, and the population of *B* cells increased at a rate proportional to the number of *A* cells previously described as thus; $\frac{dA}{dt}=0$ and $\frac{dB}{dt}=k_{1}A.$

where *A* = represents the number of *A* cancer stem cells (CSCs), *B* = represents the number of *B* tumour cells, and $k_{1}$ = the rate that *A* stem cells are diving *B* cells were terminal, so they did not divide and were produced indefinitely. A refined model [21] is as follows;

$\begin{matrix} \frac{dB}{dt}=k_{1}A\left( 1-\frac{A}{S} \right) \end{matrix}$ (3)

$\begin{matrix} \frac{dB}{dt}=k_{1}A\left( \frac{A}{S} \right)\left( 1-\frac{A}{M_{1}} \right) \end{matrix}$ (4)

$\begin{matrix} \frac{dH}{dt}=k_{2}H\left( 1-\frac{H}{M_{2}} \right) \end{matrix}$ (5)

based on the assumption that all cells grow logistically, where the parameters and their descriptions are listed in Table 2 below:

Table 2: Description of the parameters in equation

| **Parameter** | **Description** |
| --- | --- |
| *A* | Number of cancer stem cells |
| *B* | Number of tumour cells |
| *H* | Number of healthy cells vulnerable to radiation |
| $k_{1}$ | Rate that *A* stem cells are dividing |
| $k_{2}$ | Rate that *H* cells are growing |
| S | Desired number of *A* stem cells in the tumour |
| $M_{1,2}$ | Carrying capacities of *B* and *H*, respectively |
| d | Death rate of *B* cells |

It has been assumed that the initial conditions are established to depict the growth of a tumour from its initiation, such that $A(0)=A_{0}$, $B(0)=B_{0}$, and $H(0)=H_{0}$to describe the tumour at the point immediately after the first *A* cell was created. This implies initial conditions $A_{0}=1,$ $B_{0}=0,$ and $H_{0}=M_{2}$.

Since the goal of the research was to describe the effects of radiation therapy on cancer with a mathematical model, then the differential equation model here only considered radiation delivery as a constant as per the proposed model in [2] as follows:

$\begin{matrix} \frac{dB}{dt}=k_{1}A\left( 1-\frac{A}{S} \right)-r_{1} \end{matrix}$ (6)

$\begin{matrix} \frac{dB}{dt}=k_{1}A\left( \frac{A}{S} \right)\left( 1-\frac{A}{M_{1}} \right)-dB-r_{2} \end{matrix}$ (7)

$\begin{matrix} \frac{dH}{dt}=k_{2}H\left( 1-\frac{H}{M_{2}} \right)-r_{3} \end{matrix}$ (8)

where $r_{1,2,3}$ are the respective effects of radiation.

This mathematical model is referred to as the radiation treatment model. The assumption here is that cancer must grow large enough to be noticed before treatment may begin. It becomes more reasonable to begin radiation treatment when the cell populations are on the verge of approaching their carrying capacities. The model is an effective illustration of radiation treatment.
